# Two copper(II) compounds derived from tetrazole carboxylates for chemodynamic therapy against hepatocellular carcinoma cells

**DOI:** 10.3389/fchem.2022.915247

**Published:** 2022-08-29

**Authors:** Xinya Shi, Yulan Gu, Chuandan Wan, Xin Jiang, Lei Shen, Litao Tan, Yujie Zhong, Dengfeng Zou

**Affiliations:** ^1^ Changshu No. 2 People’s Hospital, Changshu, Jiangsu, China; ^2^ Central Laboratory of Changshu Medical Examination Institute, Changshu, China; ^3^ School of Pharmacy, Guilin Medical University, Guilin, China; ^4^ Department of Materials Engineering, Changshu Institute of Technology, Changshu, China

**Keywords:** Cu(II), tetrazole carboxylate, human hepatocellular carcinoma cells, chemodynamic therapy, *in vitro*

## Abstract

Two Cu(II) compounds based on tetrazole-carboxylate ligands, [Cu(phtza)_2_(H_2_O)_2_]∙3H_2_O (**1**) and [Cu(atzipa)_2_]∙2H_2_O (**2**) (phtza = 2,2'-(5,5'-(1,3-phenylene)bis(2*H*-tetrazole-5,2-diyl))diacetate, atzipa = 3-(5-amino-1*H*-tetrazol-1-yl)isopropanoic anion), were designed and synthesized by hydrothermal reactions. The X-ray diffraction results show that the two compounds show two-dimensional (2D) layer structures. Nanoprecipitation with 1,2-distearoyl-sn-glycero-3-phosphoethanolamine-N-[methoxy(polyethylene glycol)_-2000_] (DSPE-PEG_-2000_) contributes to the formation of the nanoparticles (NPs) with excellent water dispersity. *In vitro* study indicates that the two NPs exert considerable cytotoxicity toward human hepatocellular carcinoma cells (HepG2 and Huh7) with low half-maximal inhibitory concentration (IC_50_). However, the cytotoxicity of such NPs is negligible in normal cells (HL-7702). The cytotoxicity of these NPs was also investigated by the flow cytometry and Calcein-AM/PI (live/dead) co-stained experiments. The results promise the great potential of these NPs for chemodynamic therapy against cancer cells.

## Introduction

Cancer has already become a tremendous threat to health worldwide, following heart and cardiovascular diseases, and global mortality keeps rising (Siegel et al., 2018). Traditional therapeutic methods, for example, chemotherapy usually employs drugs, such as cisplatin (II) and doxorubicin, for the treatment of cancer (Tang et al., 2018; Song et al., 2019; Zhu et al., 2020). Normal cells may still suffer from side effects because the targeting ability of the compounds is very poor, though such drugs achieve considerable therapeutic efficacy to some extent. Therefore, designing and synthesizing anticancer drugs with specific targeting ability to avoid the side effects and enhance the therapeutic efficacy is an effective alternative to solve the problem (Fujita et al., 2014; Weiss et al., 2014; Sun et al., 2017a; Sun et al., 2017b; Yang et al., 2018; Yang et al., 2019a; Yang et al., 2019b; Yang et al., 2020; Zou et al., 2020). Tumor microenvironment (TME), usually features hypoxia and high hydrogen peroxide (H_2_O_2_) concentration, compared with that in normal tissues (Arneth, 2020; Deng et al., 2020). Chemodynamic therapy (denoted as CDT) can induce cell death by catalyzing H_2_O_2_ to generate cytotoxic hydroxyl radicals (⋅OH) through Fenton or Fenton-like reactions, typically Fe(II) or Cu(II) compounds (Fujita et al., 2014; Li et al., 2018; Yang et al., 2019c; Shen et al., 2019; Li et al., 2020). For example, Chen *et al.* designed a kind of nanosystem that is able to generate free radicals by iron pool for cancer chemodynamic therapy (H Zou et al., 2021). CDT is considered a non-invasive strategy to fight against cancer.

**SCHEME 1:**
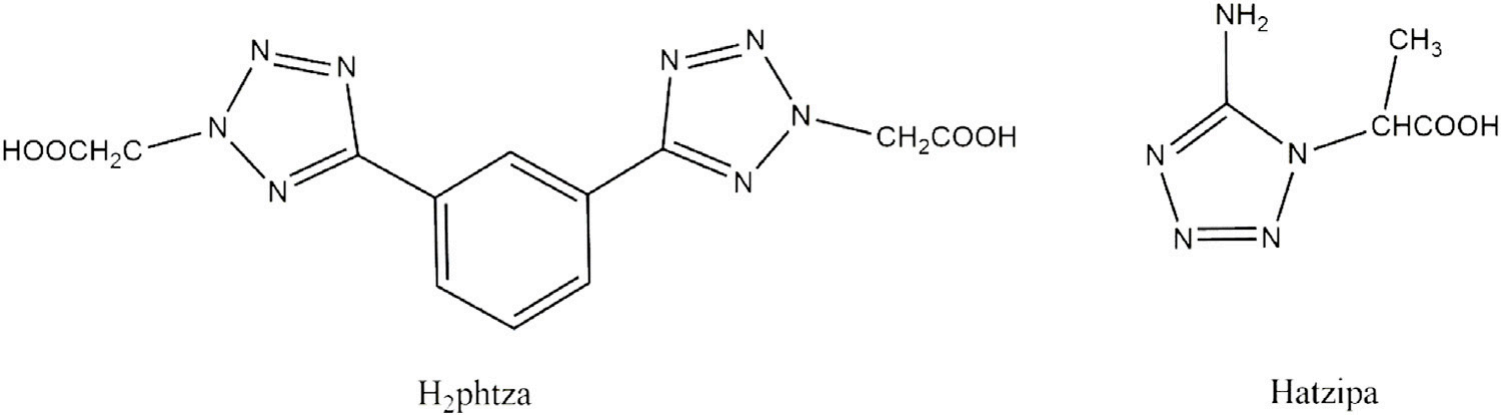
Structure illustration of H_2_phtza and Hatzipa.

Coordination compounds, a class of functional materials, are attracting increasing interest owing to not only their diverse structures but also their great potential in the field of luminescence, adsorption, and catalysis (Aromi et al., 2011; Wriedt et al., 2012; Zou et al., 2014a; Zou et al., 2015a; Du et al., 2015; Shen et al., 2016; Zhang et al., 2016; Lin et al., 2019; Zou et al., 2021). Tetrazole carboxylates are bi-functional ligands with either flexible carboxylate groups or rigid tetrazole rings that have great potential for constructing a variety of coordination architectures. These ligands with abundant nitrogen and oxygen atoms have a high possibility to show diverse coordination modes, and the CH_2_ is capable of favoring the carboxylate with flexible orientations, contributing to the formation of novel crystal structures (Zou et al., 2014b; Zou et al., 2014c; Zou et al., 2014d; Zou et al., 2015b; Dai et al., 2021). In the previous study, tetrazole-based ligands have been universally employed as multi-building blocks for the construction of novel coordination architectures and in the fields of luminescence (Zou et al., 2014b; Zou et al., 2014c; Zou et al., 2014d; Yang et al., 2014; Zou et al., 2015b; Sun et al., 2016; Zhang et al., 2016; Dai et al., 2021), catalysis (Zou et al., 2015a), and adsorption (Wriedt et al., 2012). However, the biological applications of such compounds are relatively less reported. Based on the observations, we are devoted to designing and synthesizing novel Cu(II) compounds based on two different tetrazole carboxylates, 2,2'-(5,5'-(1,3-phenylene)bis(2*H*-tetrazole-5,2-diyl))diacetic acid (Hphtza) and Hatzipa = 3-(5-amino-1*H*-tetrazol-1-yl)isopropanoic acid) (Scheme 1). As a result, [Cu(phtza)_2_(H_2_O)_2_]∙3H_2_O (**1**) and [Cu(atzipa)_2_]∙2H_2_O (**2**) were obtained by hydrothermal reactions. The nanoparticles (NPs) of the two compounds were synthesized by a nano-precipitation method with encapsulation by 1,2-distearoyl-sn-glycero-3-phosphoethanolamine-N-[methoxy(polyethylene glycol)_-2000_] (DSPE-PEG_-2000_). Further *in vitro* study suggests the two NPs show high cytotoxicity toward human hepatocellular carcinoma cells (HepG2 and Huh7). In terms of cytotoxicity, compound **1** is superior to compound **2** on HepG2 (IC_50_: 58.3 μM for compound **1** NPs and 83.6 μM for compound **2** NPs), while both complexes have IC_50_ values of 45.5 μM on Huh7 cells. Eventually, the Calcein-AM/PI (PI = 3,8-diamino-5-(3-diethylaminopropyl)-6-phenylphenanthridinium iodide) staining and flow cytometry results demonstrate that these compounds are able to induce cell apoptosis for efficient chemodynamic therapy.

## Experimental section

### Materials and apparatus

H_2_phtza and Hatzipa were prepared according to the literature (Yang et al., 2014; Sun et al., 2016). The chemicals were commercially available from Sigma-Aldrich (Shanghai). Elemental analyses (C, H, and N) were performed with a PE2400 elemental analyzer. The Fourier transform infrared (FT-IR) spectrum was measured on the Thermo NICOLET 380 instrument as a KBr disk (4,000–400 cm^−1^). The UV-vis spectra were obtained from a UV-3600 spectrometer (Shimadzu, Japan).

### Preparation and characterization of compounds 1 and 2

A mixture of Cu(NO_3_)_2_·3H_2_O (241 mg, 0.1 mmol) and H_2_phtza (165 mg, 0.05 mmol) in a mixture of water (2 ml) and ethanol (2 ml) was sealed in a stainless steel container and heated at 120°C for 24 h. After cooling to room temperature, the solution was filtered for evaporation. Blue crystals were obtained. Elemental analyses Calcd. for C_12_H_18_CuN_8_O_9_ (%): C, 29.91; H, 3.74; N, 23.25. Found (%): C, 29.73; H, 3.77; N, 23.40. IR (KBr, cm^−1^): 3,022 (w), 2,901 (w), 2,245(w), 1,623 (m), 1,577 (m), 1,521 (s), 1,124 (vs), 1,088 (s), 935 (w), 868 (m), 725 (m), and 623 (s).

The synthesis procedure for compound **2** is similar to that of **1** except that H_2_phtza has been replaced by Hatzipa (79 mg, 0.05 mmol). Elemental analyses Calcd. for C_36_H_32_Cl_2_CuN_8_O_10_ (%): C, 23.33; H, 3.88; N, 34.00. Found (%): C, 23.43; H, 3.82; N, 34.50. IR (KBr, cm^−1^): 3,440 (m), 3,088 (w), 2,933 (w), 1,635 (w), 1,568 (m), 1,494 (s), 1,385 (m), 1,114 (vs), 925 (w), 855 (w), 738 (m), and 629 (s).

### Crystal structure determination

Suitable single crystals of **1** and **2** were selected for collection of intensity data on a Bruker SMART APEX II CCD diffractometer using a *ϕ*-*ω* scan mode at 291 K for compound **1** and 296(2) K for compound **2** [monochromate Mo *K*_*α*_ radiation (*λ* = 0.71073 Å)]. SADABS was applied for multi-scan absorption corrections of all intensity data. The structures were solved by direct methods, and SHELXTL software was used to refine *F* ^2^ by full-matrix least squares procedures (Sheldrick, 2008). All hydrogen atoms were fixed in calculated positions and refined isotropically. The crystallographic data of compounds **1** and **2** are listed in Table 1. The selected bond lengths and angles are shown in Table 2. The hydrogen bond parameters are listed in Supplementary Table S1.

**TABLE 1:** Selected crystal data of compounds **1** and **2**.

Compound	1	2
Empirical formula	C_12_H_18_CuN_8_O_9_	C_8_H_16_CuN_10_O_6_
Formula mass	481.88	411.85
Crystal system	Monoclinic	Monoclinic
Space group	*P*2_1_ */c*	*P*2_1_ */c*
*a* (Å)	16.736 (3)	5.1139 (14)
*b* (Å)	13.127 (2)	12.271 (3)
*c* (Å)	8.5308 (13)	12.598 (3)
*α* (°)	90.00	90
*β* (°)	90.407 (4)	106.139 (9)
*γ* (°)	90.00	90
*V* (Å^3^)	1874.1 (5)	759.4 (3)
*Z*	4	2
*T* (K)	291	293 (2)
Dcalcd (g.cm^−3^)	2.074	1.668
*μ* (mm^−1^)	1.23	1.49
Reflections collected	12,577	3,934
Unique reflections (*R*_int_)	4,291 (0.0604)	1,333 (0.041)
R^[^ ^a^ ^]^ and *w*R^[^ ^b^ ^]^	0.0525 and 0.1404	0.041 and 0.1261
GOF^[^ ^c^ ^]^	0.974	1.015
Δ/ρmax (e/Å^3^)	0.70	0.54
Δ/ρmin (e/Å^3^)	-1.134	-0.41

a R = ||F_o_|-|F_c_|/|F_o_|.

b Rw = {*w*(F_o_^2^-F_c_^2^)^2^/w(F_o_^2^)^2^}^1/2^.

c GOF = {*w*((F_o_^2^-F_c_^2^)^2^)/(n-p)}^1/2^, where *n* = number of reflections and *p* = total number of parameters refined.

**TABLE 2:** Selected bond distances (Å) and angles (°) for compounds **1** and **2**.

**Compound 1**			
Cu(1)–O(1)	1.936 (3)	Cu(1)–O(3A)	1.977 (3)
Cu(1)–O(6)	1.957 (3)	Cu(1)–O(4B)	2.210 (3)
Cu(1)–O(5)	1.973 (3)	O(1)–Cu(1)–O(6)	90.75 (14)
O(1)–Cu(1)–O(5)	90.87 (14)	O(6)–Cu(1)–O(5)	175.83 (16)
O(1)–Cu(1)–O(3A)	174.93 (13)	O(6)–Cu(1)–O(3A)	87.39 (14)
O(5)–Cu(1)–O(3A)	90.68 (14)	O(1)–Cu(1)–O(4B)	92.62 (12)
O(6)–Cu(1)–O(4B)	96.22 (15)	O(5)–Cu(1)–O(4B)	87.54 (14)
O(3A)–Cu(1)–O(4B)	92.27 (12)		
**Compound 2**			
Cu(1)–O(1)	1.943 (3)	N(4)–Cu(1D)	1.999 (3)
Cu(1)–O(1A)	1.943 (3)	N(4B)–Cu(1)–N(4C)	180.0
Cu(1)–N(4B)	1.999 (3)	C(1)–O(1)–Cu(1)	120.6 (3)
Cu(1)–N(4C)	1.999 (3)	C(4)–N(4)–Cu(1D)	132.6 (3)
O(1)–Cu(1)–O(1A)	180.0 (2)	N(4)–N(4)–Cu(1D)	120.8 (3)
O(1)–Cu(1)–N(4B)	87.56 (13)	O(1A)–Cu(1)–N(4B)	92.44 (13)
O(1)–Cu(1)–N(4C)	92.44 (13)	O(1A)–Cu(1)–N(4C)	87.56 (13)
Cu(1D)–N(4)–C(4)–N(1)	177.0 (3)		

Symmetry code: For **1**: A: *x*+1, −*y*+3/2, *z*+1/2; B: *x*+1, *y*, *z*+1; C: *x*−1, −*y*+3/2, *z*−1/2. For **2**: A: −x+1, −y, −z+1; B: x, −y+1/2, z+1/2; C: −x+1, y−1/2, −z+1/2; D: −x+1, y+1/2, −z+1/2.

### Hydroxyl generation by Fenton-like reaction

10 μg mL^−1^ MB, 8 mM H_2_O_2_, and 0.5 mM compounds **1** or **2** NPs were allowed to stand at 37°C for 30 min. The OH-induced MB degradation was monitored by the absorbance change at 665 nm.

### Synthesis of compounds 1 and 2 nanoparticles

The nanoparticles (NPs) were prepared by nanoprecipitation with DSPE-PEG_-2000_. A mixture of DSPE-PEG_-2000_ (10 mg) and compound **1** or **2** (2 mg) was dissolved in tetrahydrofuran (THF) under ultrasound (100 W). Then, such a solution was injected into 5 ml distilled water under ultrasound. After the mixture was sonicated in an ultrasonic washer for 3–5 days, THF was removed by purging nitrogen gas at room temperature. The solution was stored at 4°C for characterization and cytotoxicity experiment.

### Cell culture and cytotoxicity assay

Hepatocellular carcinoma cell lines (HCC, including HepG2 and Huh7) and human normal cells (HL-7702) were available from the Shanghai Institute of Biochemistry and Cell Biology, Chinese Academy of Sciences (CAS). HepG2 and Huh7 cells were cultured in DMEM (Biosharp; Biosharp Life Sciences) with 10% FBS (Biosharp; Biosharp Life Sciences) at 37°C with 5% CO_2_.

Cell viability was determined using a Cell Counting Kit. HepG2 cells (2 × 10^3^/well), Huh7 cells (2 × 10^3^/well), and HL-7702 (2 × 10^3^/well) were seeded into 96-well plates. The cells were cultured with the NPs of compounds **1** and **2** for 24 h, respectively. Cytotoxicity was evaluated using the Cell Counting Kit-8 (CCK-8, Abbkine Scientific). CCK-8 was added to the medium and incubated with the cells for 2 h. Then, the absorbance was measured on a microplate reader. The cell viability was calculated as follows:

$$\text{Relative}\;\text{cell}\;\text{viability}\left(\%\right)=\left(A_{treatment}/A_{control}\right)\times 100\%$$

where A_treatment_ = mean absorbance of the medium from cells incubated with NPs containing complex 1 or 2 and A_control_ = mean absorbance of the medium incubated without NPs of non-treated cells. The half-maximal inhibitory concentration (IC_50_) was calculated using SPSS 25.0 software.

The MTT assay was repeated three times.

### Flow cytometry

Cell apoptosis of the nanoparticles was detected by flow cytometry. Generally, HepG2 cells (2 × 10^5^/well) and Huh7 cells (2 × 10^5^/well) were seeded in 6-well plates and cultured for 24 h. Then, the cells were co-cultured with NPs of compound **1** or **2** for 24 h at the concentration of IC_50_ and 2 × IC_50_, respectively. Cell apoptosis was detected by the FITC Annexin V Apoptosis Detection Kit I on the flow cytometry (Attune NxT, Invitrogen by Thermo Fisher Scientific). The flow cytometry was repeated three times.

### Live/dead co-staining by Calcein-AM and PI

HepG2 and Huh7 (both concentration ∼2 × 10^4^/well) were seeded in 24-well plates. In this experiment, we set up control groups, IC_50_ groups and 2 × IC_50_ groups, respectively. Calcein AM is a cell-permeant dye that can be used to determine cell viability in most eukaryotic cells. In live cells, the non-fluorescent calcein AM is converted to a green-fluorescent calcein after acetoxymethyl ester hydrolysis by intracellular esterases. Calcein AM is used to stain live cells (green channel), while PI is used to stain dead cells (red channel). In the control group, the cells were cultured, and then the cells were treated with compounds **1** and **2** NPs for 24 h at the concentration of IC_50_ and 2 × IC_50_, respectively. Then, the medium was discarded, and the cells were washed with PBS three times. The cells were co-stained with a Living/Dead cell double staining kit (Sigma-Aldrich (Shanghai) Trading Co., Ltd.) with the concentration of calcein-AM at 2.5 μM and PI 4.5 μM, respectively. After 20 min, the cells were washed with PBS three times. The photographs were captured on an inverted fluorescence microscope, Olympus FV1000 confocal microscope.

### Statistical analysis

All numeric data are expressed as mean ± s.d. unless otherwise indicated. The significance between the two groups was analyzed by a two-tailed Student’s t-test. Statistical analysis was performed using GraphPad Prism 6.0. *p* values of less than 0.05 were considered significant (**p < 0.01 and ***p < 0.01).

## Results and discussions

### Description of crystal structures of [Cu(phtza)_2_(H_2_O)_2_]∙3H_2_O (1) and [Cu(atzipa)_2_]∙2H_2_O (2)

**FIGURE 1:**
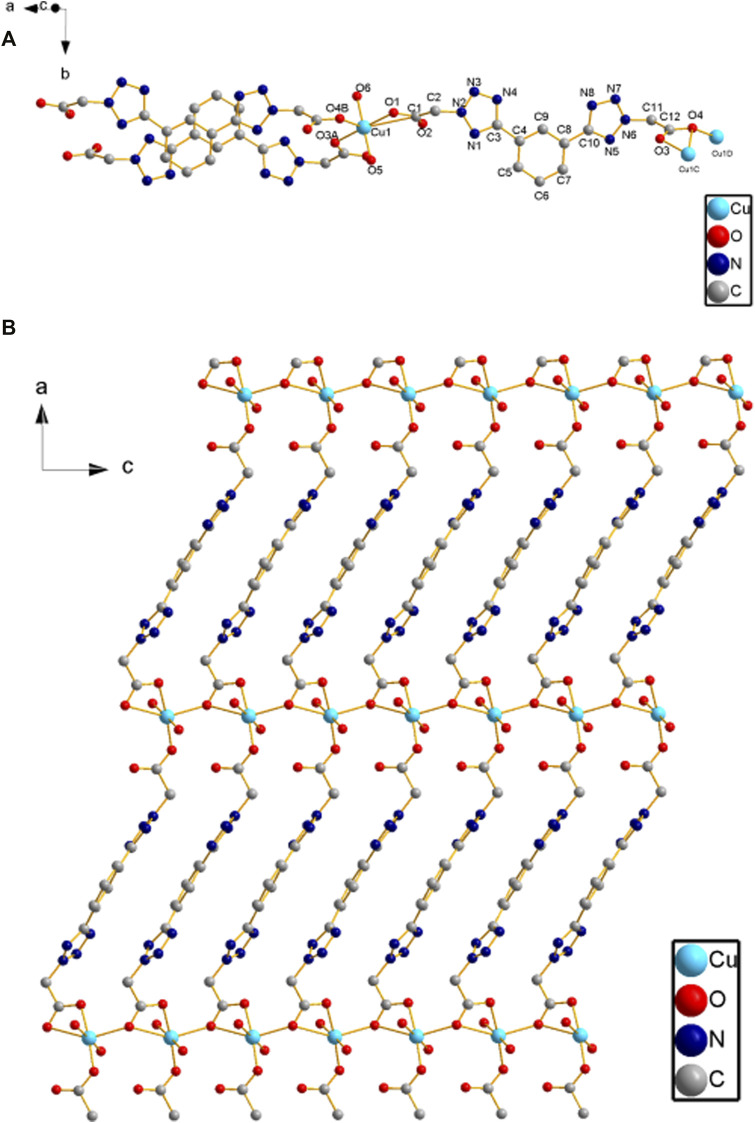
**(A)** Coordination environment of Cu(II) in compound **1**. **(B)** 2D layer structure of compound **1** parallel to the *ac* plane. Hydrogen atoms are omitted for clarity.

**FIGURE 2:**
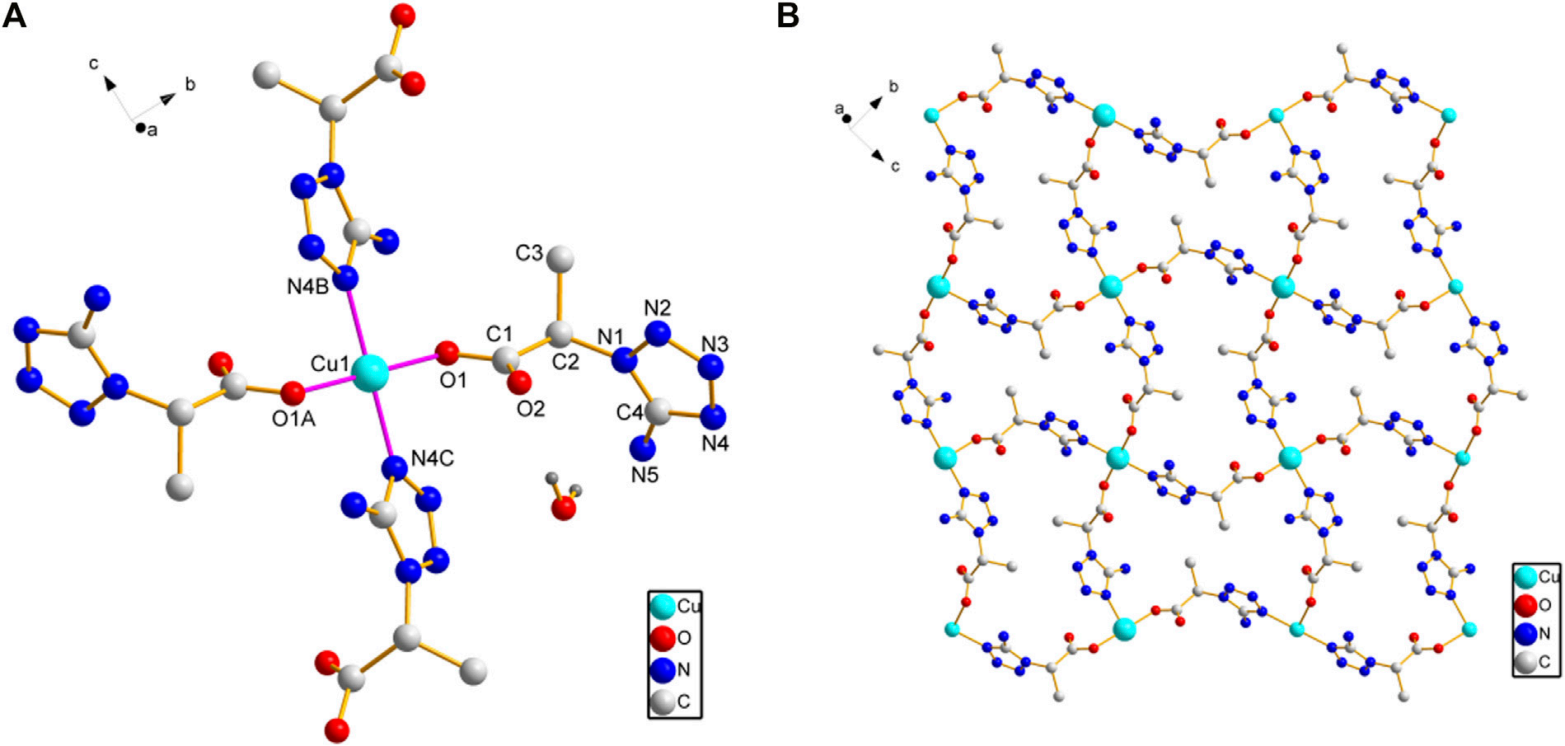
**(A)** Coordination environment of Cu(II) in compound **2**. **(B)** 2D layer structure of compound **2** parallel to the *bc* plane. Hydrogen atoms are omitted for clarity.

X-ray crystallography results reveal that both compounds **1** and **2** crystallize in monoclinic lattice with space group *P*2_1_/*c.* Compound **1** is made up of one Cu(II), two phtza^−^ anions, two coordinated water, and three lattice water molecules, while compound **2** consists of one Cu(II), two atzipa^−^ anions, and two lattice water (Figures 1A, 2A). The phtza ligand is a tetra-dentate to bridge the adjacent Cu(II) centers by two carboxylate oxygen atoms in a *μ*_1,1,3_-COO or a *μ*_1_-COO mode, respectively, to generate a two-dimensional (2D) layer structure parallel to the *ac* plane (Figure 1B). In contrast, atzipa in compound **2** adopts a bridging mode by the tetrazole nitrogen atoms one carboxylate oxygen, thereby also forming a 2D layer structure parallel to the *bc* plane (Figure 2B). The Cu-O bond distance of compound **1** is 1.943 Å and that of compound **2** ranges from 1.936 to 2.210 Å, while the Cu-N bond distance of compound **1** is 1.999 Å. Adjacent 2D layers are connected together by hydrogen bonding to generate a three-dimensional (3D) supramolecular structure (Supplementary Figures S1, S2; Supplementary Table S1). Compared with the previously reported mononuclear [Cu(2-pytzipa)_2_(H_2_O)_2_]·2H_2_O (Zhai et al., 2017) (2-pytzipa = 5-(2-pyridyl)tetrazole-2-isopropanoic anion) in which 2-pytzipa only adopts the N (pyridyl) and N(tetrazole) chelating mode, the coordination modes of phtza and atzipa are more complicated. 3-pytza in [Cu(3-pytza)_2_(H_2_O)]_n_·2nH_2_O (Zou et al., 2014e) shows a N(pyridyl) and O (COO^−^) bridging mode, which is similar to that of atzipa in compound **2**. All the nitrogen atoms in phtza in compound **1** are all uncoordinated.

**FIGURE 3:**
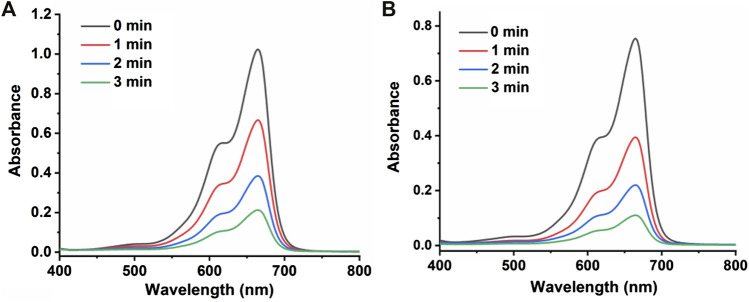
MB degradation in the presence of H_2_O_2_ and **(A)** compound **1** NPs and **(B)** compound **2** NPs.

Since methylene blue (MB) can be degraded by hydroxyl radicals, the hydroxyl radicals’ generation ability of compounds **1** and **2** NPs was investigated by recording the absorbance of methylene blue (MB) in the presence of H_2_O_2_ (Figure 3). The absorbance of MB continued to decrease because of the generation of hydroxyl radicals catalyzed by the Fenton-like reaction of compounds **1** or **2** NPs and H_2_O_2_. In addition, compound **1** NPs are superior to compound **2** NPs in terms of catalysis.

### Cytotoxicity and flow cytometry

**FIGURE 4:**
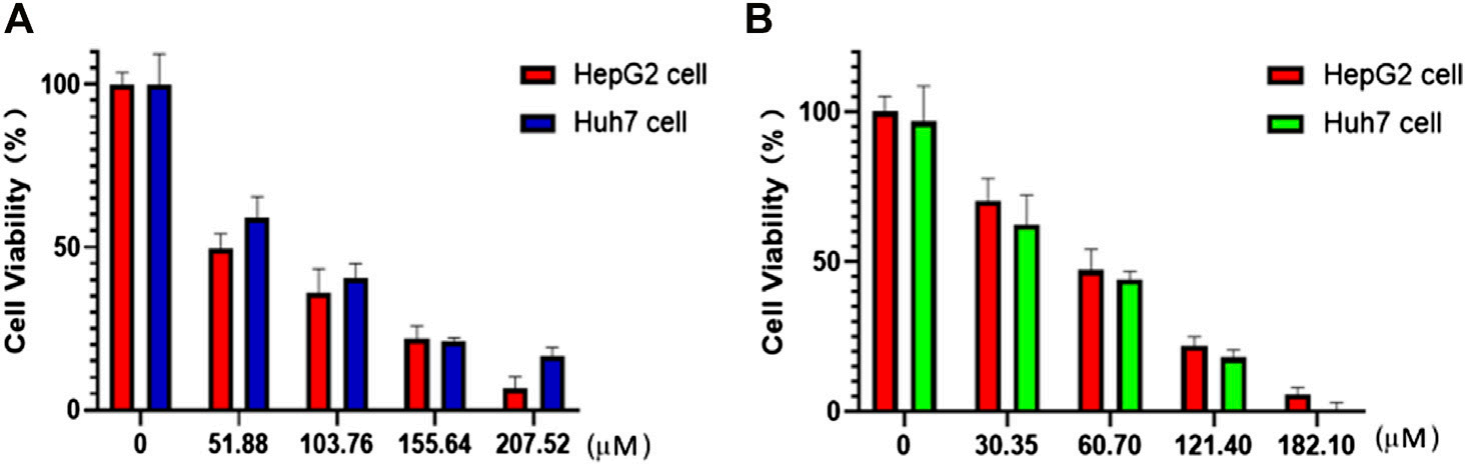
*In vitro* CCK8 assay of HepG2 and Huh7 cells treated with **(A)** compound **1** NPs and **(B)** compound **2** NPs.

The nanoparticles (NPs) of compounds **1** and **2** were prepared by nanoprecipitation with DSPE-PEG_-2000_ due to their excellent dispersity and stability in aqueous solution. Two different human hepatocellular carcinoma cells, HepG2 and Huh7, were chosen to investigate the chemodynamic therapeutic efficacy of NPs of compounds **1** and **2** by the Cell Counting Kit-8 (CCK-8) assay. CCK-8 allows sensitive colorimetric assays for the determination of cell viability in cell proliferation and cytotoxicity assays. The results showed concentration-dependent cell death after two types of liver cancer cells were treated with NPs of compound **1** or **2** under different concentrations (Figure 4). With the increase of the concentrations, the viability of HepG2 and Huh7 cells tends to gradually reduce. For HepG2 cells, the IC_50_ values of compounds **1** and **2** NPs are 58.3 and 83.6 μM, respectively. For Huh7 cells, the IC_50_ of both NPs is 45.5 μM. Both compounds have strong inhibitory effects on the cell activity of the two cell lines. Compound **2** is superior to compound **1** on HepG2 cells, while it is parallel to compound **1** on Huh7 cells in terms of cytotoxicity. Huh7 cells are more sensitive to the NPs containing complexes **1** and **2** than HepG2 cells. Compared with the relevant Cu(II) compounds based on tetrazole ligands, the cytotoxicity of the two compounds is lower than that of [Cu(atzpa)_2_] and [Cu(pytzipa)_2_] (Zhang et al., 2021) but higher than that of [Cu(2-pytzipa)_2_(H_2_O)_2_]·2H_2_O (Zhai et al., 2017) (Table 3). The cytotoxicity of these NPs is very low toward healthy hepatocytes because the cell viability remained high (Supplementary Figures S3, S4).

**TABLE 3:** Comparison of cytotoxicity of Cu(II) compounds based on tetrazole ligands.

Compound	Cell lines	IC_50_ (μM)	References
[Cu(atzpa)_2_]	HeLa	10.9	Zhang et al. (2021)
[Cu(pytzipa)_2_]	HeLa	6.7	Zhang et al. (2021)
[Cu(2-pytzipa)_2_(H_2_O)_2_]·2H_2_O	HeLa	70.0	Zhai et al. (2017)

atzpa = 3-(5-amino-tetrazol-1-yl)-propionic anion), pytzipa = 2-(5-pyridin-3-yl-tetrazol-2-yl)-propionic anion), and 2-pytzipa = 5-(2-pyridyl)tetrazole-2-isopropanoic anion.

**FIGURE 5:**
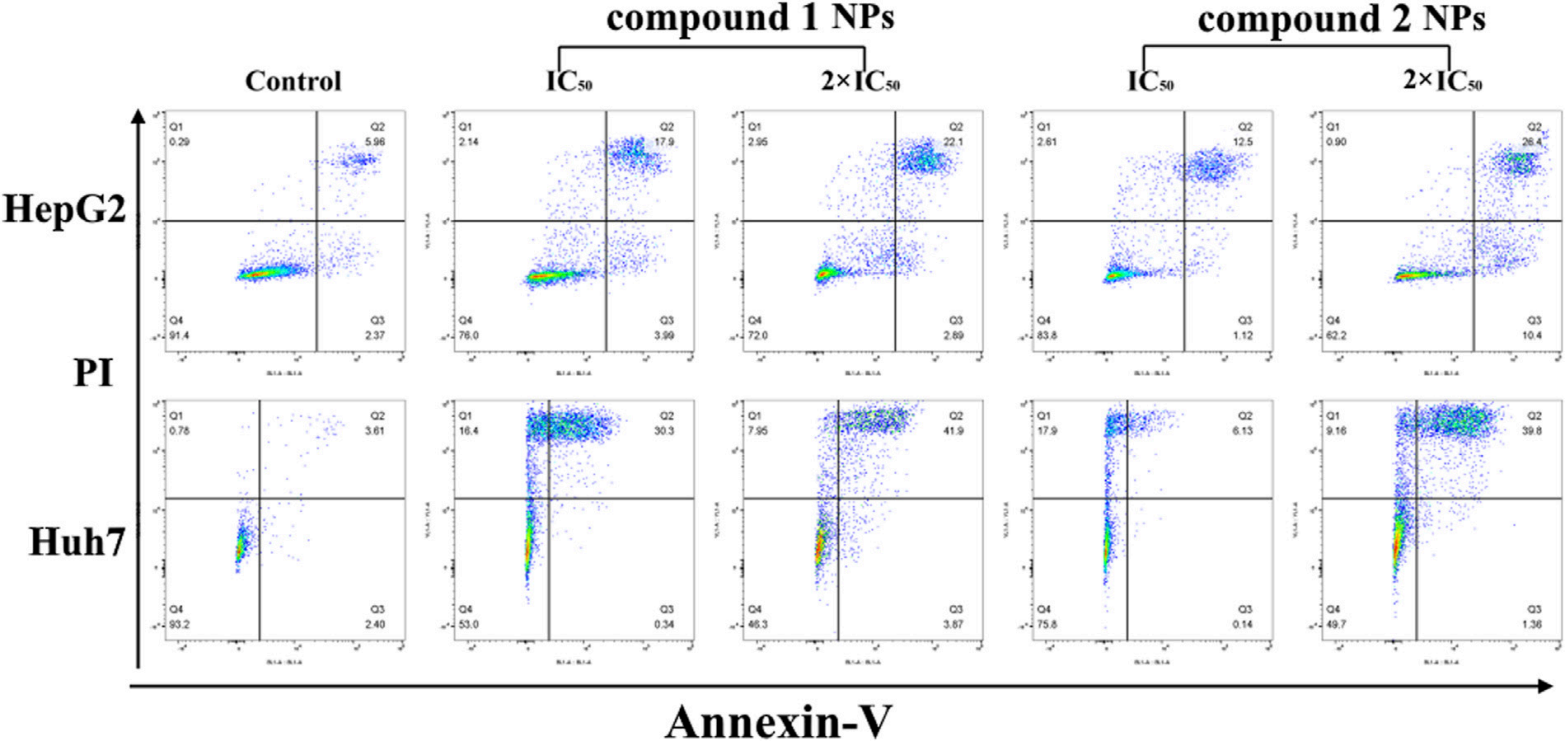
Flow cytometry results of compounds **1** and **2** NPs on HepG2 and Huh7 under different concentrations.

The cytotoxicity of compounds **1** and **2** NPs was further confirmed by flow cytometry, a technique used to detect and measure the physical and chemical characteristics of a population of cells or particles. The results show that the cell apoptotic rates of HepG2 and Huh7 cells in the groups treated with compounds **1** and **2** were significantly increased but markedly different. In particular, all the groups of 2 × IC_50_ were significantly higher than IC_50_ groups, and the apoptosis rates (Q2 + Q3) of the control groups were rather low (Table 4). For the control group, both HepG2 and Huh7 cell viability are very high (87.27% for HepG2 and 93.99% for Huh7), while concentration-dependent cell viability was observed for those treated with compounds **1** or **2** NPs. These results demonstrated that the compound **1** and compound **2** NPs could induce and promote cell apoptosis of Huh7 and HepG2 cells (Figure 5). Late apoptosis of the cells treated with compound **1** NPs was detected, while both necrosis and late apoptosis were detected for the cells incubated with compound **2** NPs.

**TABLE 4:** Apoptosis rate of compounds **1** and **2** NPs on HepG2 and Huh7 cells.

	Control (%)	Compound 1 NPs		Compound 2 NPs	
		IC_50_ (%)	2 × IC_50_ (%)	IC_50_ (%)	2 × IC_50_ (%)
HepG2	9.94 ± 2.79	17.27 ± 3.99	20.16 ± 4.87	15.46 ± 1.59	43.33 ± 5.66
Huh7	6.01 ± 0.00	25.09 ± 5.31	42.06 ± 3.61	15.79 ± 11.09	33.04 ± 8.19

### Live/dead co-staining by Calcein-AM and PI

**FIGURE 6:**
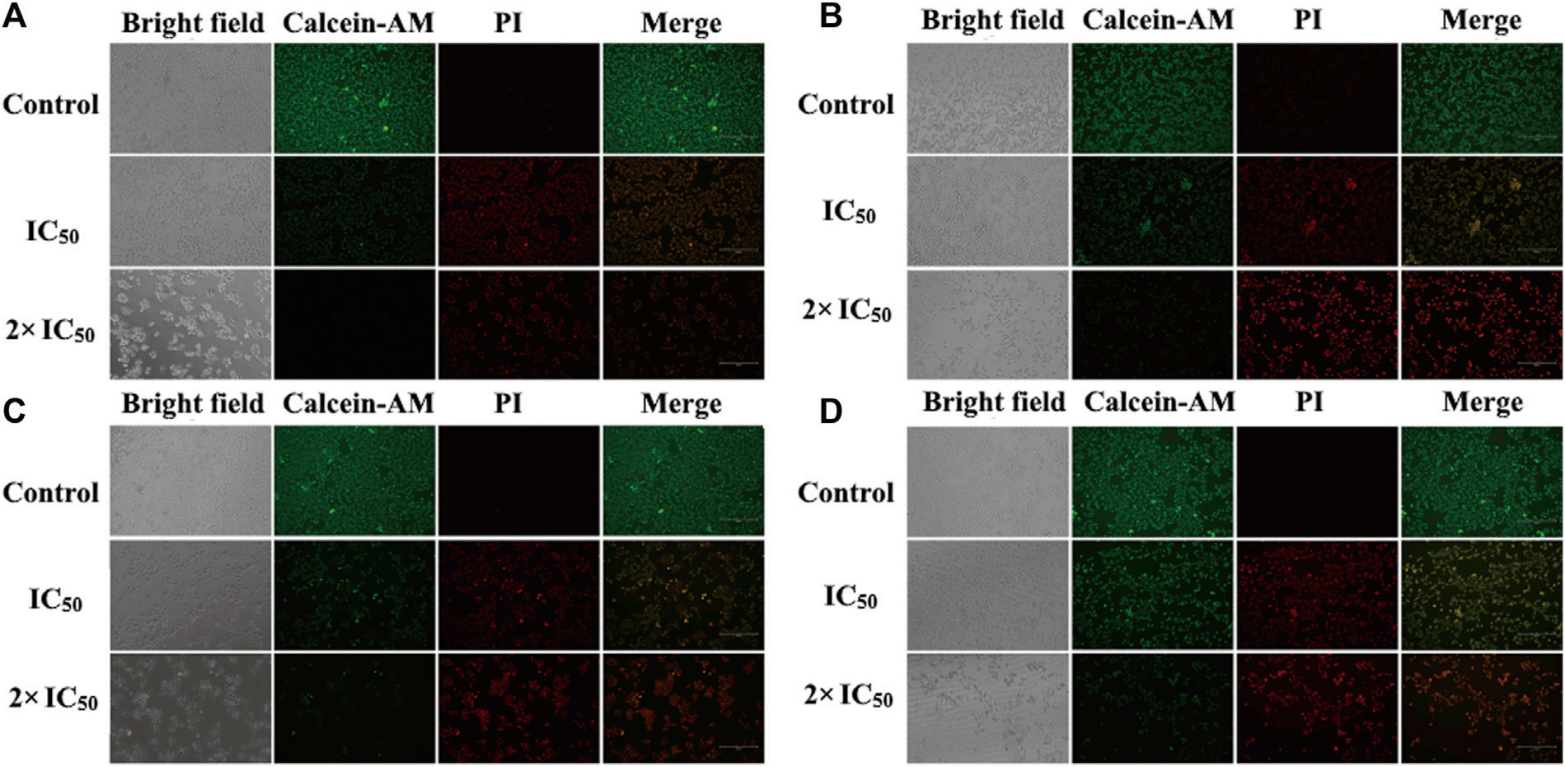
*In vitro* calcein AM and PI co-staining with compound **1** NPs **(A)** HepG2 cells and **(B)** Huh7 cells. Compound **2** NPs **(C)** HepG2 cells and **(D)** Huh7 cells.

**FIGURE 7:**
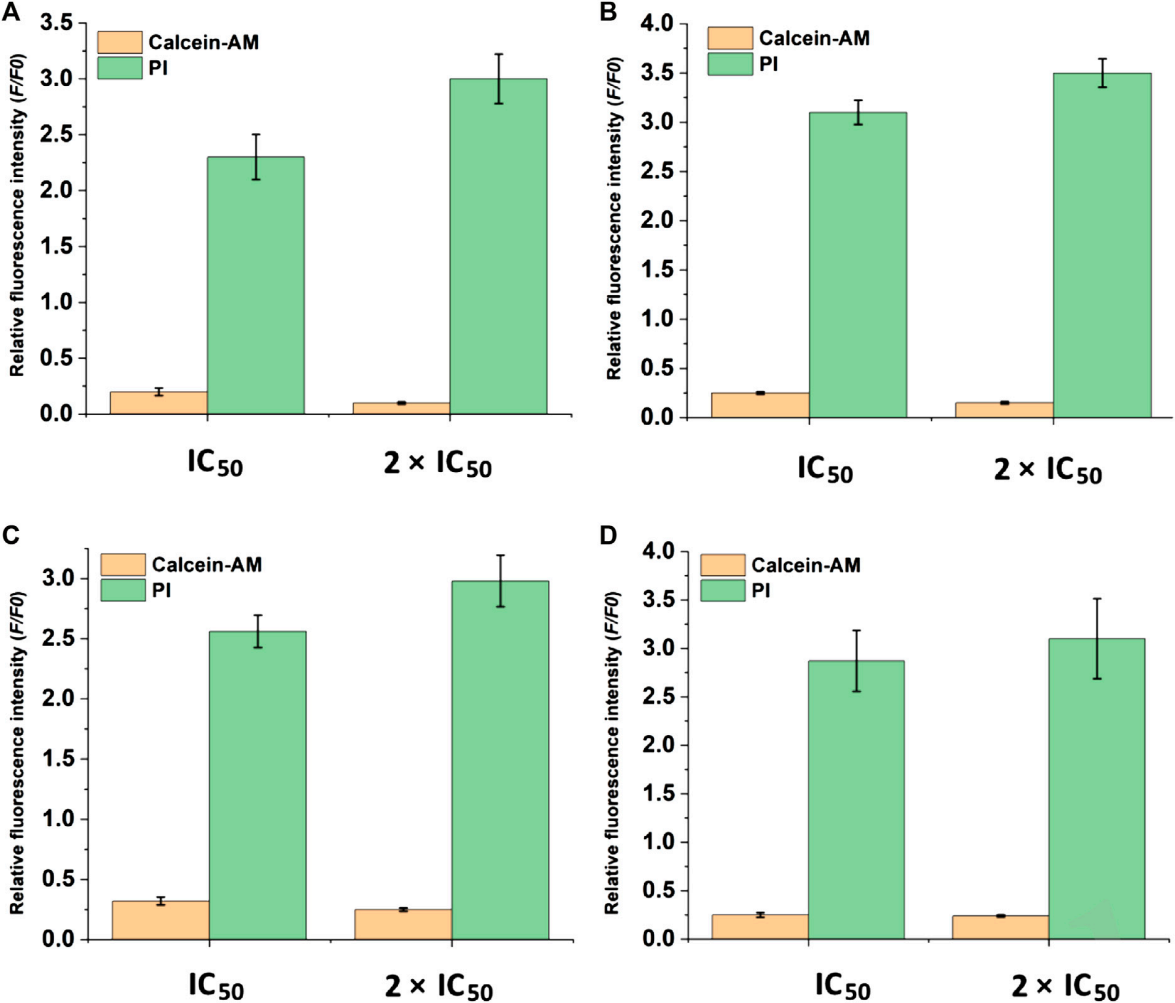
Quantification of fluorescence intensity of calcein AM and PI-stained cells. **(A)** HepG2 cells and **(B)** Huh7 cells treated with compound **1** NPs. **(C)** HepG2 cells and **(D)** Huh7 cells treated with compound **2** NPs.

After the discovery of the inhibitory and toxicity effects of compounds **1** and **2** on the HCC cells. The cell viability was investigated by the live/dead co-staining (Figure 6), where living cells can be stained with calcein AM to show green fluorescence and dead cells with PI to exhibit red fluorescence. The cells in control groups expressed large areas of strong green fluorescence, and the faint red fluorescence was almost negligible, indicating that all cells were alive. However, in the groups treated with compound **1** and compound **2** NPs, a large number of cells were stained with red fluorescence. Cell death is more obvious in the treatment groups with a concentration of 2 × IC_50_ than that with IC_50_ (Figure 6). The quantitative fluorescence intensity of the calcein AM and PI also indicates that compounds 1 and 2 NPs can induce cell apoptosis by CDT (Figure 7).

## Conclusion

To conclude, two new Cu(II) tetrazole carboxylates were designed and prepared for chemodynamic therapy against HCC. These compounds with new 2D structures are capable of catalyzing H_2_O_2_ to form cytotoxic hydroxyl radicals, promising their excellent cytotoxicity toward HepG2 and Huh7 cells. Nanoprecipitation with DSPE-PEG_-2000_ was used to prepare compounds **1** and **2** NPs with good water dispersity. Both the CCK-8 and Calcein-AM/PI co-staining assay confirmed the cytotoxicity of compounds **1** and **2** NPs, which was consistent with the flow cytometry results. Further study is still underway in our group to overcome the H_2_O_2_ consumption-induced shortage for enhanced chemodynamic therapy, such as H_2_O_2_ self-supply.

## Data Availability

The data presented in the study are deposited in the https://pan.baidu.com/s/1pgI7nZDVN1IXKZRmgYcPOA repository, accession number fic2.
