# Supplementary material for: Two copper(II) compounds derived from tetrazole carboxylates for chemodynamic therapy against hepatocellular carcinoma cells
Source: Front Chem. 2022 Aug 29;10:915247. doi: 10.3389/fchem.2022.915247 (PMC9467286; doi:10.3389/fchem.2022.915247)
Supplement: Supplementary file 1 [file DataSheet1.docx]

**Two copper(II) compounds derived from tetrazole carboxylates for chemodynamic therapy against hepatocellular carcinoma cells**

Xinya Shi^a^, Yulan Gu^a^*, Chuandan Wan*^b^, Xin Jiang^c^, Lei Shen^d^, Litao Tan^d^, Yujie Zhong^d^, Dengfeng Zou^c^*

1. *Changshu No.2 People’s Hospital, Changshu, 215500, Jiangsu, P.R. China. Email:* [*guyulan@263.net*](mailto:guyulan@263.net)
2. *Central Laboratory of Changshu Medical Examination Institute, Changshu, 215500, P.R. China. Email:* [*cd_wan@163.com*](mailto:cd_wan@163.com)
3. *School of Pharmacy, Guilin Medical University, Guilin, 541004, Guangxi, P.R. China. Email:* [*zdf1226@126.com*](mailto:zdf1226@126.com)
4. *Department of Materials Engineering, Changshu Institute of Technology, Changshu, 215500, P.R. China.*

**Table S1. Hydrogen-bonding Geometry (Å and º) for compounds 1-2**

| D—H···A | D—H | H···A | D···A | D—H···A |
| --- | --- | --- | --- | --- |
| **Compound 1** |  |  |  |  |
| O(3)-H(3D) ···O(2) | 0.85 | 2.49 | 2.855(5) | 110 |
| O(3)-H(3E) ···O(1)#2 | 0.85 | 2.44 | 2.283(5) | 173 |
| O(3)-H(3E) ···O(2) | 0.85 | 2.57 | 2.885(5) | 103 |
| N(5)-H(5A) ···O(3)#1 | 0.86 | 2.21 | 2.969(5) | 147 |
| N(5)-H(5B) ···O(2)#3 | 0.86 | 2.43 | 3.126(5) | 138 |
| C(2)-H(2A) ···O(3)#1 | 0.98 | 2.55 | 3.296(5) | 133 |

**Compound 2**

O(5)-H(5B) ···O(8) 0.85 1.88 2.720(6) 169

O(5)-H(5C) ···O(2) #1 0.85 1.95 2.800(5) 178

O(6)-H(6B) ···O(9) 0.85 2.01 2.679(5) 134

O(6)-H(6C) ···N(4) #4 0.85 2.23 2.806(5) 125

O(7)-H(7B) ···N(1) 0.85 2.18 2.981(9) 158

O(7)-H(7C) ···N(3) #3 0.85 2.58 3.407(10) 166

O(8)-H(8A) ···O(7) 0.85 2.27 2.705(10) 112

O(8)-H(8B) ···O(1) #2 0.85 2.22 2.902(5) 137

O(9)-H(9B) ···N(8) #4 0.85 2.15 2.960(6) 159

O(9)-H(9C) ···N(5) #1 0.85 2.37 3.209(5) 171

C(2)-H(2A) ···O(7) #5 0.97 2.40 3.294(9) 153

C(11)-H(11B) ···O(3) #2 0.97 2.40 3.158(5) 134

Symmetry codes:

For **1**:＃1: -1+x,y,z; #2: 1+x, y,-z; #3: −1+x,1/2 −y,−1/2+z.

For **2**: #1: 1+x, 3/2-y, 1/2+z; #2: x, 3/2-y, -1/2+z; #3: x, 3/2-y, 1/2+z; #4: 1-x, 1-y, 1-z; #5: 1-x, -1/2+y, 3/2-z.


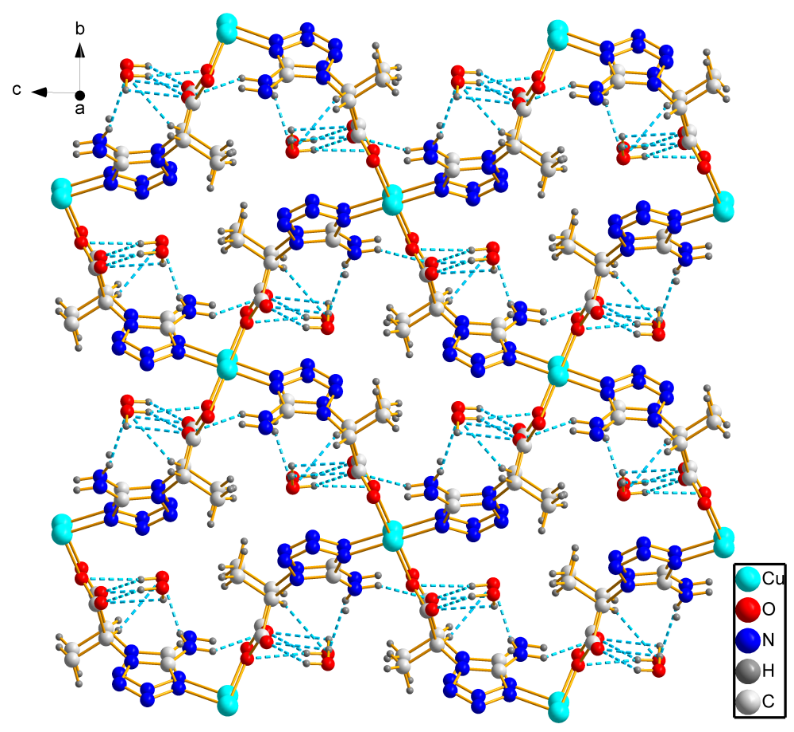


Figure S1 3D supramolecular structure of compound **1** formed by hydrogen bonds.


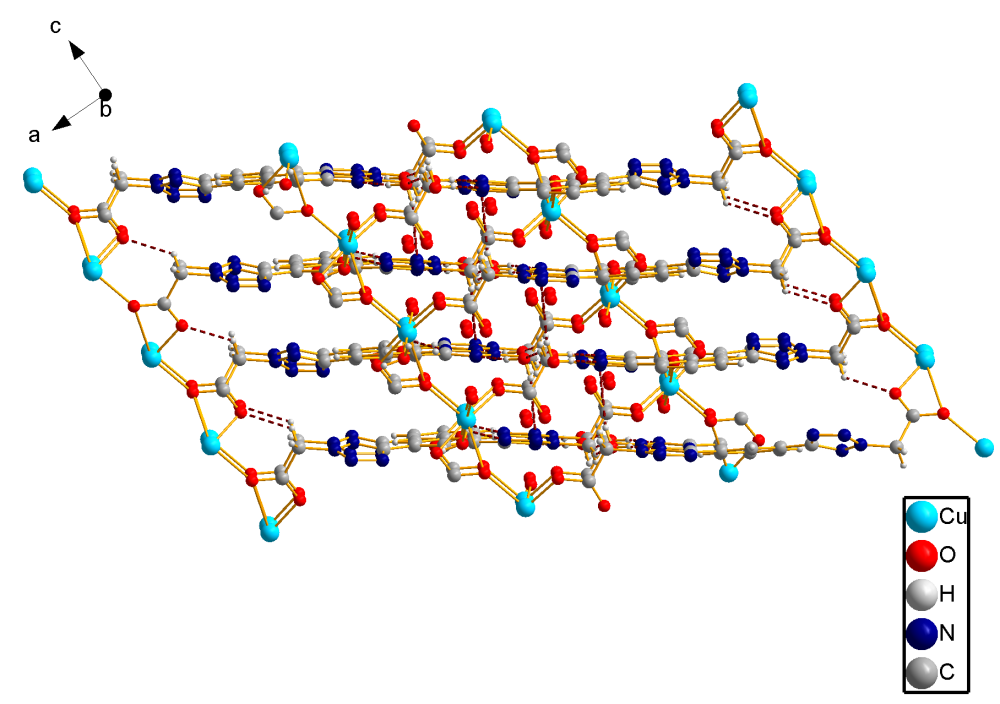


Figure S2 3D supramolecular structure of compound **2** formed by hydrogen bonds.


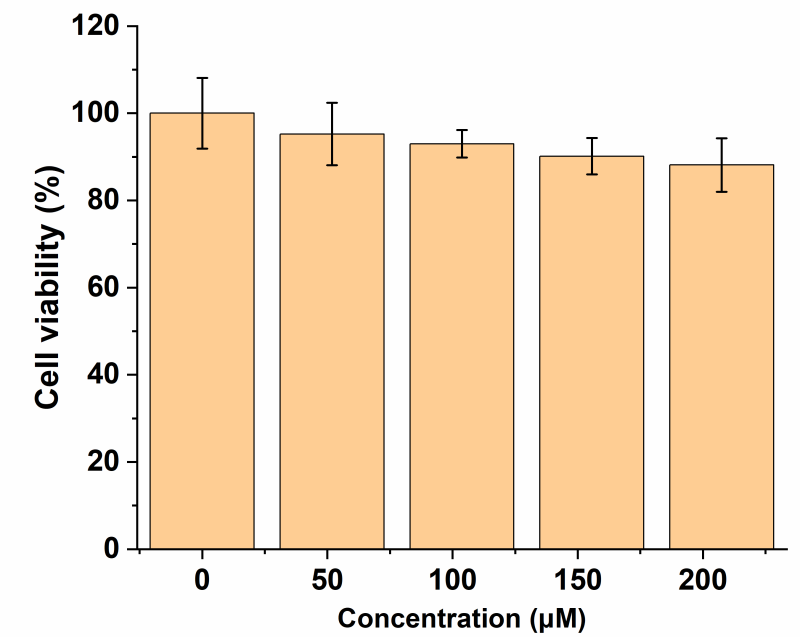


Fig. S3 Cell viability of compound **1** NPs on HL-7702 cells.


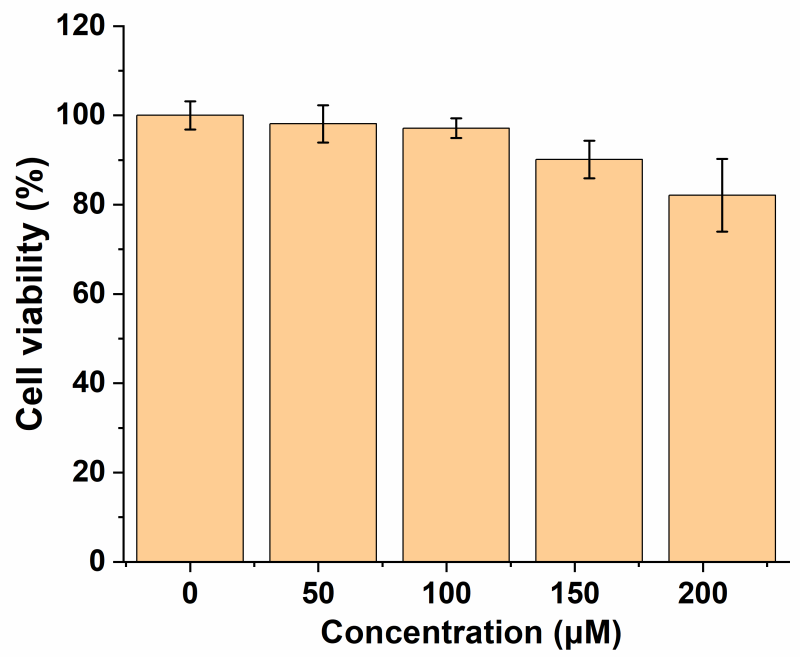


Fig. S4 Cell viability of compound **2** NPs on HL-7702 cells.
